# Fluorosomes: Fluorescent Virus-Like Nanoparticles that Represent a Convenient Tool to Visualize Receptor-Ligand Interactions

**DOI:** 10.3390/s130708722

**Published:** 2013-07-08

**Authors:** Daniela Wojta-Stremayr, Winfried F. Pickl

**Affiliations:** 1 Christian Doppler Laboratory for Immunomodulation, Vienna 1090, Austria; 2 Center for Pathophysiology, Infectiology and Immunology, Institute of Immunology, Medical University of Vienna, Borschkegasse 8A, Vienna 1090, Austria

**Keywords:** virus-like nanoparticles, fluorosomes, fluorescent dyes, receptor-ligand interactions

## Abstract

Viruses are the smallest life forms and parasitize on many eukaryotic organisms, including humans. Consequently, the study of viruses and viral diseases has had an enormous impact on diverse fields of biology and medicine. Due to their often pathogenic properties, viruses have not only had a strong impact on the development of immune cells but also on shaping entire immune mechanisms in their hosts. In order to better characterize virus-specific surface receptors, pathways of virus entry and the mechanisms of virus assembly, diverse methods to visualize virus particles themselves have been developed in the past decades. Apart from characterization of virus-specific mechanisms, fluorescent virus particles also serve as valuable platforms to study receptor-ligand interactions. Along those lines the authors have developed non-infectious virus-like nanoparticles (VNP), which can be decorated with immune receptors of choice and used for probing receptor-ligand interactions, an especially interesting application in the field of basic but also applied immunology research. To be able to better trace receptor-decorated VNP the authors have developed technology to introduce fluorescent proteins into such particles and henceforth termed them fluorosomes (FS). Since VNP are assembled in a simple expression system relying on HEK-293 cells, gene-products of interest can be assembled in a simple and straightforward fashion—one of the reasons why the authors like to call fluorosomes ‘the poor-man's staining tool’. Within this review article an overview on virus particle assembly, chemical and recombinant methods of virus particle labeling and examples on how FS can be applied as sensors to monitor receptor-ligand interactions on leukocytes are given.

## 1. Virus Like-Nanoparticles—Just Empty Shells?

Virus-like nanoparticles (VNP) are self-assembling, non-replicating and non-infectious particles resembling in size and shape the parent viruses they are derived from. Over-expression of viral structural proteins in different primary cells or permanent cell lines including cells of bacterial, yeast, plant, insect and mammalian origin, leads to the formation of VNP representing highly-organized particulate structures of defined size [1–5]. So far, VNP of a wide variety of different viruses belonging either to the RNA or DNA orders, and either enveloped or non-enveloped, have been created and induced by recombinant expression in producer cell lines of either single or multiple viral capsid proteins (reviewed in [6]).

How is VNP budding from producer cells induced by single or multiple capsid proteins? In fact, budding of viral particles is generally induced by the self-assembling tendency of viral structural proteins. These proteins carry distinct determinants for self-assembly and have the tendency to form bended spherical structures upon aggregation with each other. The size of the particles formed during this process is dictated by the biophysical properties of the respective core proteins, which allow a certain degree of bending. Of note, alteration of a given core sequence also causes variations in particle size and shape [7–9]. The end product of the budding process is a stable, often geometrically sophisticated three-dimensional structure, which characteristically differs between virus families. Enveloped C-type viruses comprising retroviruses including HIV, which use the plasma membrane of the infected host cell as a ‘second coat’, display in addition to the self-assembling properties of their core protein(s) some peculiarities associated with the budding process. In order to get enveloped by the plasma membrane, their core proteins become typically post-translationally modified by covalent attachment of myristic acid to their N-terminal glycine residue [10,11]. Proteins with linear lipid modifications in the form of long fatty acids have the tendency to insert themselves into special regions of the plasma membrane characterized by an ordered lipid environment and commonly referred to as lipid rafts or membrane microdomains [12,13]. These modifications function as membrane anchors for the newly translated core proteins, which otherwise would freely float around within the ocean of cytoplasmic proteins. Moreover, this mechanism also concentrates the core proteins at the cytoplasmic aspect of the plasma membrane, which can be appreciated as electron-dense patches in ultrastructural analyses [14]. This is the way newly synthesized core proteins find each other, start to interact with each other and eventually form new particles. The high core protein concentration at the inner aspect of lipid rafts facilitates physical interactions between neighboring proteins so that core protein intrinsic factors can come into play and promote their further aggregation and stabilization. In addition, a positively charged stretch of amino acids within the N-terminal region of most core proteins, provided by a cluster of basic residues brought about by an accumulation of arginines and lysines, promotes their additional association with the inner aspect of the plasma membrane [15–19]. These residues most probably contribute to electrostatic interactions with negatively charged head-groups of acidic phospholipids at the inner leaflet of the plasma membrane [20,21]. Protein-sequence intrinsic features together with the posttranslational lipid modifications increase the stability of the overall interaction of core proteins with the plasma membrane [22–24].

Why and how enveloped viral particles eventually bud from the plasma membrane is still enigmatic. One hypothesis to explain budding claims that the spherical structure formed by core protein aggregation is strong enough to coat itself with membrane lipids and finally to pinch-off the cell (pulling force of the virus particle) (reviewed in [25]). Another hypothesis proposes that in the last phase of budding, newly formed particles reside on stalks, which might be responsible for particle coating, maturation and release (pushing force of host-encoded proteins) (reviewed in [25]). In fact, disruption of distinct regions within Gag sequences of retroviruses, now commonly referred to as late domains (L-domains) have been shown to be of critical importance during the latest steps of budding [26], since they actively interact with host cell molecules involved in the generation of cellular vesicles, which bud away from the host cell. Along those lines several proteins belonging to the endosomal sorting complex required for transport (ESCORT) and providing binding-pockets for L-domains of retroviruses have been identified [27–29]. Thus host cell intrinsic mechanisms of particle formation and release might in fact become usurped by the nascent viral proteins making virus budding something like a ‘joint effort’ between the host cell and viral factors not only regarding replication of viral genetic information but also during the particle formation and release process.

Other types of VNP not budded from producer cells have been described in the past. In fact, VNP can be generated *in vitro* by benefiting from the self-assembling tendency of viral capsid proteins under certain environmental conditions, commonly referred to as chemical self-assembly processing. This approach circumvents purification steps usually required to remove contaminating material of viral and non-viral origin from producer cell-derived *in vivo* generated VNP (reviewed in [30]). Such directly assembled virus particles laid the ground [5,31,32] for the generation of the already commercialized HPV vaccine [33,34] as well as for the development of experimental vaccines against e.g., hepatocellular carcinoma based on HBV particles [35], group A streptococcus based on murine polyomavirus [36], human parvovirus [37] and porcine circovirus type 2 [38]. Apart from that application, self-assembling VNP are currently being tested as drug-delivery vehicles. A very promising approach in that respect are bacteriophage MS2-based particles delivering microRNAs to target cells with high efficiency by virtue of a transactivator of transcription (Tat) cell-penetrating peptide fusion mechanism [39–41]. In fact, the envelopment of biologically active RNAs significantly ameliorates their half-life, cellular up-take and targeting specificity. Targeted cell types involve among others myelogenous leukemia cells as well as cervical cancer cells [39,41]. In addition, classical chemotherapeutic agents such as the intercalating anthracycline doxorubicin have been successfully attached to the self- assembling rotavirus VP6 protein to target hepatic cancer cells [42].

Moreover, genetic fusion of fluorescent proteins to self-assembling hepatitis B virus capsid proteins allowed the synthesis of fluorescent capsid nanoparticles (FCNPs) showing high *in vitro* and *in vivo* stability when compared to monomeric fluorescent proteins, thus representing potential tools for *in vivo* optical imaging [43]. Magnetic particles incorporated into self-assembling M13 bacteriophages and equipped with a peptide targeting sequence have been successfully applied to detect prostate cancer tumors by magnetic resonance imaging *in vivo* [44].

## 2. Making the Obvious Visible

Replication of viruses as obligatory intracellular parasites strictly depends on the host cells, providing the necessary machineries for genome replication and protein synthesis, as well as the assembly and budding of the viral offspring. Prior to host cell infection a highly specific interaction between molecules on the surface of the virus (spike proteins, ligand) and (an) interaction partner(s) of the host cell (receptor) is a *conditio sine qua non*. Visualization of virus-host cell interactions, *i.e.*, receptor-ligand interactions, was in early virological studies accomplished by utilizing electron microscopy. Along those lines Dales showed already in 1962 attachment of two mammalian viruses to host cells by means of electron microscopy [45]. However, electron microscopy bears the major disadvantage that cells to be analyzed have to become fixed and thus cannot be applied to living cells making kinetic studies difficult. The development of fluorescent microscopy and the ever growing variety of available fluorescent dyes allowed to directly visualize not only the interaction between entire viruses and host cells, but also to localize and track virus proteins at the subcellular level. This formed a solid basis for the development of methods for real-time single-virus tracking within living cells and permitted to follow the fate of individual viral particles [46]. Moreover, fluorescent proteins enabled the researchers to *in vivo* localize and trace viral particles [47,48]. Furthermore, flow cytometry enlarged the possible applications for fluorescent viral particles, e.g., by making direct quantification of virus-host interactions possible. In fact, several approaches to label viral particles have been devised over the last decades, which in general can be divided into either methods relying on chemical coupling of fluorophores to the virus surface or genetic fusion of fluorescent proteins to viral structural or non-structural proteins. In the following section a comprehensive overview on labeling methods will be provided with a special focus on fluorescent virus-like nanoparticles, so called fluorosomes (FS).

## 3. Techniques Applied for the Chemical Labeling of Viral Particles

**Figure 1.**
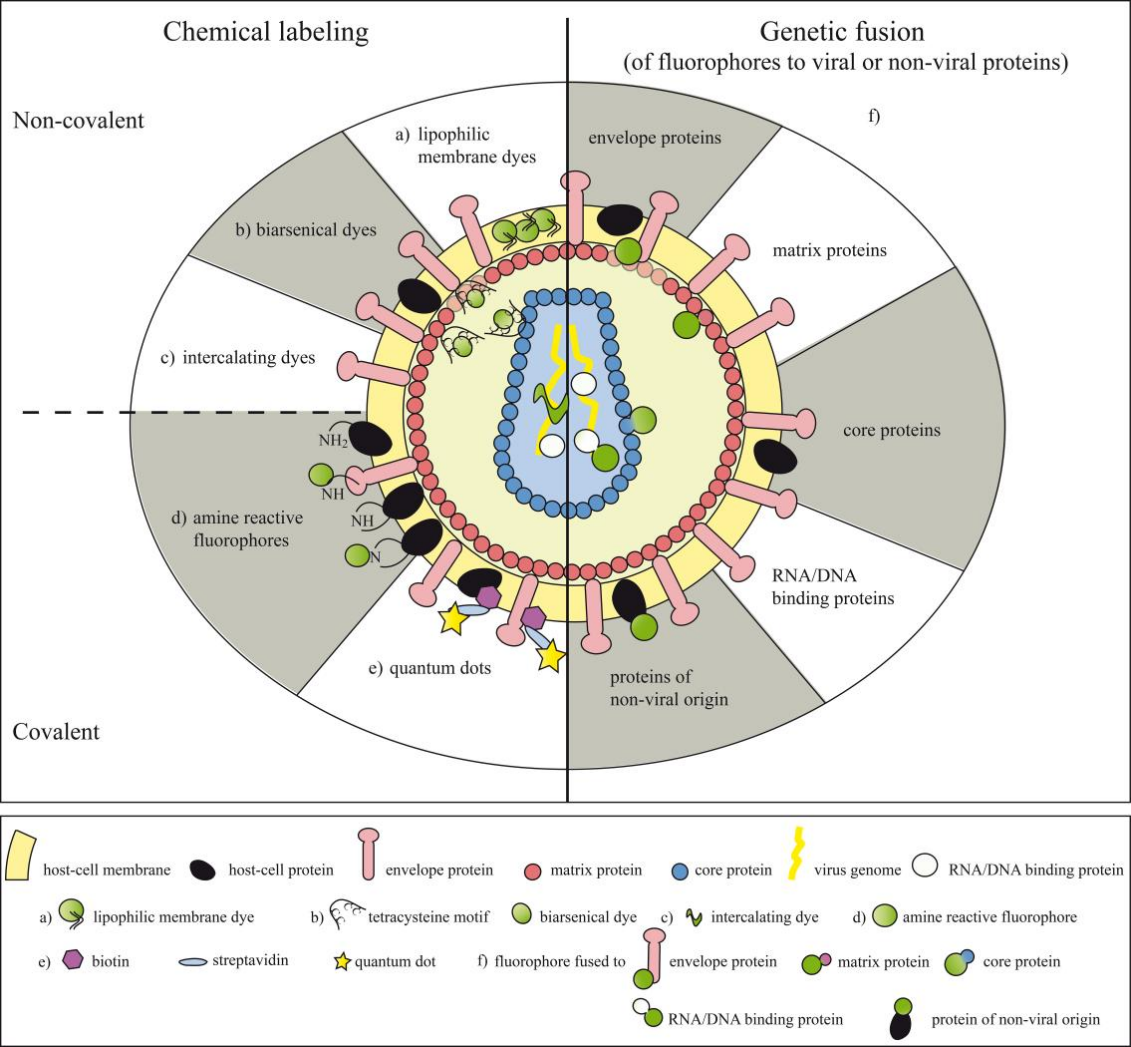
Methods applied for the labeling of viral particles. Viral particles can be labeled by chemical labeling methods (covalent or non-covalent) or by fusion of fluorophores to proteins integrated into the viral particle. Non-covalent labeling of viral particles can be achieved by (**a**) lipophilic membrane dyes inserting themselves into the host-cell membrane, (**b**) biarsenic dyes binding to tetracysteine motifs introduced into viral proteins or (**c**) intercalating dyes interacting with the viral genome. Alternatively, (**d**) amine reactive fluorophores can be covalently attached to proteins present on the surface of the viral membrane. (**e**) Attachment of streptavidin coupled quantum dots can be achieved by biotinylating target structures of interest. (**f**) Fluorophores can be genetically fused to proteins of viral and non-viral origin.

Chemical compounds can be either coupled non-covalently or covalently to viral particles, making the labels more or less stably associated with their target structures (Figure 1 and Table 1). Chemical labeling can also be performed after the virus particles have already assembled.

## 4. Non-Covalent Attachment of Fluorophores

### 4.1. Lipophilic Membrane Dyes and Dyes for the Viral Genome

Enveloped viruses, which are enveloped into the plasma membrane of the host cell, can be non-covalently stained with classical membrane dyes like PKH67 or PKH26. The PKH dyes were named after their inventor Paul Karl Horan and were patented in the late 1980s [85]. PKH dyes consist of a fluorescent dye attached to long lipophilic side chains, which are readily inserted into biological membranes. Staining of viral particles with PKH67 or PKH26 to study virus-host interactions has been proven a very quick and simple method without the need of cumbersome purification steps following the staining procedure [49].

A very elegant method to study membrane fusion between viruses and their corresponding host cell membranes applies to the lipophilic dyes octadecyl rhodamine B chloride (R18) and carbocyanines (e.g., Dil, DiO, DiD, DiR), which can be incorporated into biological membranes [86,87]. If R18 or carbocyanines are present at high concentrations in close proximity—e.g., on the surface of viral particles—their fluorescence becomes largely quenched. Upon fusion with another cellular membrane, e.g., plasma or endosomal membranes, the dyes are diluted by lateral diffusion on the membrane of the target cell and an increase in fluorescence can be readily detected [50-59].

In order to study the fate of the viral genome upon virus entry intercalating agents like [Ru(phen)^2^(dppz)]^2+^ staining the genome of viruses can be utilized to visualize cellular uptake and subcellular distribution of viruses [60].

### 4.2. Biarsenical Dyes

The development of biarsenical compounds associated with fluorophores, like fluorescein arsenical helix binder (FlAsH) and resurfin arsencial helix binder (ReAsH), which interact with proteins containing a tetracysteine motif (tc-tag) (-CCxxCC-) [88] allowed non-covalent labeling of proteins with very high affinities (Kd 10^−11^) [89]. Both the tagged molecules and the biarsenical compounds are non-fluorescent when unbound. Upon binding of the biarsenical molecule to the tetracysteine motif, they start to emit a strong fluorescence signal. Intr oduction of the very small amino acid motif into viral structural proteins by means of genetic modification allowed labeling and subsequent subcellular localization of different types of viruses (reviewed in [61-68,90]). In contrast to other chemical labeling methods, the use of tetracysteine motifs in combination with biarsencial compounds permitted to obtain kinetic information on protein synthesis by sequential labeling with FlAsH (green) and ReAsH (red) [68,91]. Moreover, the combination of a biarsenical dye, staining matrix protein (M protein), with a fluorescent phosphoprotein (P protein) fusion protein, staining the ribonucleoprotein (RNP) allowed to investigate the early events upon VSV infection and the different fates of viral core protein and RNP after uncoating of double fluorescent virus particles [61]. Two major drawbacks of using biarsenical dyes relate to the necessity for genetic modification of viral proteins, which stands in marked contrast to other chemical labeling methods and their tendency to cause high background fluorescence due to unspecific binding to cysteine motifs of cellular origin [92]. However, the relatively small genetic modifications required for the introduction of the tetracysteine motif into viral proteins served as an attractive alternative to fusion of viral proteins to entire fluorescent proteins [61,66].

## 5. Covalent Attachment of Fluorophores

In order to achieve covalent attachment of chemical compounds to viruses or viral particles, the potential of amine-reactive groups of different fluorophores, e.g., of fluorescein, rhodamine or Alexa dyes has been explored. Fluorophores containing amine-reactive groups like N-hydroxysuccinimidyl (NHS) reactive ester groups or isothiocyanate (NCS) reactive groups can be coupled to primary amines of viral proteins thus allowing to visualize early virus-host interactions (Table I). In addition, the use of pH-sensitive dyes (e.g., CypHer5) enlarges the repertoire of applications for labeled viral particles by making it possible to sense differences in the hydrogen ion concentrations of the compartments the viruses or parts of them are shuttling through, e.g., after infection [50]. Moreover, the recently developed fluorescent semiconductor nanocrystals, so called quantum dots (Qdots) might overcome some of the spectral drawbacks of organic fluorophores, like narrow bandwidth of excitation, overlapping emission spectra and poor photostability. Qdots consist of a cadmium selenide (CdSe) core coated by a zinc sulfide (ZnS) shell [93,94]. Qdots absorb photons over a broad range of wavelengths but their emission spectrum is very confined and can be varied depending on the size and composition of the nanocrystal core [95–99]. Qdots can be either covalently conjugated to sulfhydryl or amino groups of proteins of interest by attachment of reactive amino or carboxylic groups into the organic coating of Qdots [100]. Alternatively, non-covalent coupling can be achieved using distinct proteins with highly positive charges such as avidin, which have been shown to tightly adsorb to chemically modified Qdots [101]. This offers the possibility to link proteins of interest using the avidin/biotin system with high affinity to Qdots. In fact, this system has been successfully utilized to attach Qdots to viral particles [80–83]. Alternatively, colloidal clustering in the presence of the cationic polymere polybrene can be used to form complexes between virus particles and Qdots [84]. Due to its instability this labeling method seems to be rather inferior when compared to the above described covalent labeling procedures. In order to visualize virus-host interactions quantum dots have been successfully attached to e.g., human T cell leukemia virus type 1 (HTLV-1) [80], hepatitis A virus (HAV) [83], murine norovirus (MNV-1) [83], influenza A virus [82], Moloney murine leukemia virus (MoMLV) [84] and infectious hematopoietic necrosis virus (IHNV) [81].

A clear advantage of using chemical labeling procedures to stain viruses or virus particles is the fact that no precious space within the viral genome coding for the fluorophore has to be sacrificed, which is *per se* restricted due to size limitations of the packaging machinery. Moreover, without the need of genetic modifications of virus proteins no risk regarding proper protein folding subsequent to particle assembly has to be taken into account. Nevertheless, it is also evident that chemical labeling, might lead to denaturation of important viral proteins thus impacting on their function. Moreover, random attachment of fluorophores to sites important for virus-host interactions might interfere with virus binding and infectivity. Large chemical fluorophores might even compromise virus biology when attached in the neighborhood of important interaction sites due to steric hindrance. However, due to the plasma membrane coat of enveloped viruses, chemical dyes do not get access to the viral capsid of some viruses. Thus upon target cell entry of such particles and following un-coating, they lose their specific fluorescence which was only associated with the virus envelope. Moreover, since the information for the fluorescence activity of such particles is not encoded within the viral genome, the viral offspring of chemically labeled viruses lose their fluorescence restricting the application of chemically labeled viruses primarily to the investigation of early events during viral infection. In addition, chemical labeling of whole particles has to remain restricted to one fluorophore, *i.e.*, one color. In order to circumvent these limitations, labeling of viruses or virus-like nanoparticles (VNP) by genetic fusion of structural or non-structural proteins to fluorescent proteins of cnidarian origin (e.g., GFP, RFP, YFP or codon-optimized variants thereof) [102–104] or so called “fruit colors” (e.g., mCherry) [105] has been devised.

Besides the above-discussed methodologies for labeling virus particles other cell surface membrane sensors have been created and explored on a cellular background in the past. Among them aptamer sensors [106], gold particles with unique surface plasmon resonance properties, sensors based on cleavage of fluorescent substrate by specific enzymes as well as cleavage dependent bioluminescence inducing sensors [107]. The future will show whether these technologies, which proofed to be useful to study the interaction of cells with their environment, will also become adapted to investigate virus-host interactions.

## 6. Generation of Fluorescent Viral Particles by Genetic Fusion of Fluorophores to Proteins of Viral or Non-Viral Origin

A variety of different viruses belonging to the family of both DNA or RNA viruses (enveloped or non-enveloped) have been fluorescently tagged by fusion of genes encoding virus and fluorescent proteins (Figure 1 and Table 2). In such instances each particle carries the genetic information for the fluorescently labeled viral proteins, thus each generation of virus offspring will also expresses the tagged proteins. Consequently and in contrast to chemical labeling, which allows to study only the early events upon viral infection, genetic labeling allows to explore much larger aspects of the viral life cycle. The concurrent tagging of several viral proteins with fluorescent proteins emitting light of different wavelengths allowed to follow the fate of such viral proteins upon virus infection but also during virus assembly [108–111]. In order to avoid interferences between large fluorescent proteins and particle formation, enveloped viruses have also been decorated with modified GFP forms, which have a high intrinsic affinity for the virus envelope. For that purpose GFP has been fused to GPI-anchor acceptor sequences [112] or membrane targeting sequences derived from proteins of non-viral origin [113,114].

Interestingly, decoration with modified forms of GFP can also be achieved after virus assembly has already been completed since GPI-anchored molecules can insert themselves spontaneously into VNP [146]. The authors termed this process ‘fluorescence molecular painting (FMP)’ [114].

In an attempt to explore the versatility of virus derived nanoparticles as reductionist membrane derived platforms to interrogate not only mere virus-host cell interactions but also to test specific receptor-ligand interactions between molecules of interest in the immune system we utilized fluorescent virus-like nanoparticles (fluorosomes), in which the matrix protein of MoMLV was fused to GFP giving rise to a MA∷GFP fusion protein [115]. The modular make-up of FS allowed us to additionally decorate VNP with various ligands or receptors and to subsequently test them for specific binding to target cells expressing the corresponding receptor-ligand structure.

## 7. Virus-Like Nanoparticles as a Novel Versatile Platform to Visualize Receptor-Ligand Interactions

**Figure 2.**
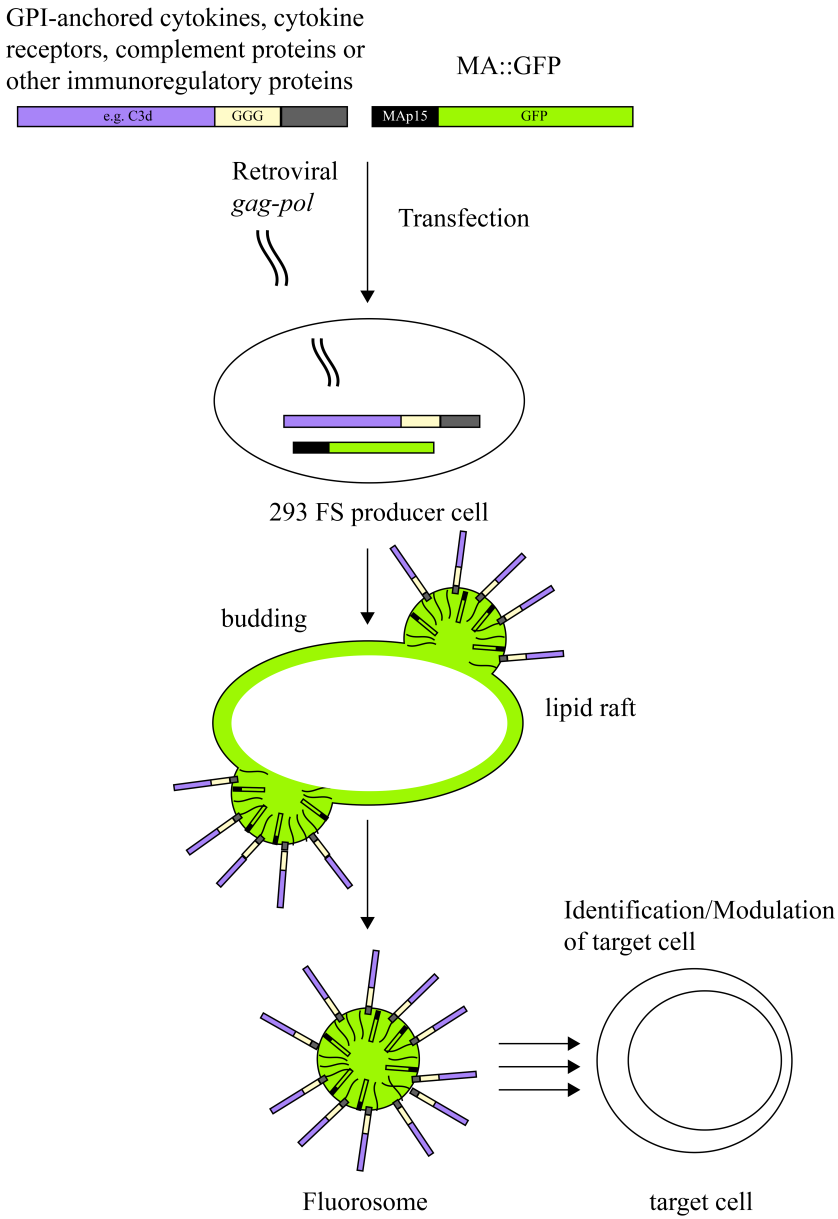
Scheme for the production of fluorosomes (FS) decorated with cytokines, cytokine receptors, complement proteins or other immunoregulatory proteins. Proteins of interest genetically fused to membrane anchors containing GPI-anchor acceptor sequences, are—upon expression in 293 cells—targeted to lipid rafts of the plasma membrane. Similarly, in order to obtain fluorescent VNP GFP fused to MoMLV matrix protein (MA) is targeted to lipid rafts within the plasma membrane. Formation of plasma membrane derived FS is induced by co-transfection of producer cells with MoMLV *gag-pol* (OGP). Lipid raft resident molecules are incorporated into the FS arising. For target cell identification or modulation either VNP can be applied directly or purified by ultrafiltration and/or ultracentrifugation.

VNP induced by core-proteins from enveloped viruses such as MoMLV represent modular platforms, which can be deliberately decorated with molecules of choice without altering the biological activity of the latter [115,147]. VNP not only incorporate heterologous viral envelope proteins (if present) but also proteins originating from the host cell if they reside in the lipid raft regions of plasma membranes of producer cells [12]. In fact, incorporation of extra- and intracellular constituents of host cell lipid rafts is a well-known phenomenon central to pseudotyping. Pseudotyping initially described the association of viral cores of one virus with the envelope proteins of a second virus, which tends to happen during mixed infections of a given target cell [148–151]. Over the last decades, pseudotyping has in fact proven to be a very useful tool for extending the host range of viral vector systems [152–154]. Thus, lipid rafts of the plasma membrane represent an important meeting point for phylogenetically different virus core and envelope proteins but also for post-translationally lipid-modified host cell proteins. Consequently, targeting of candidate molecules to lipid rafts, by e.g., fusion to a glycosyl phosphatidyl inositol (GPI)-anchor acceptor sequence, shuttles them onto the VNP surface of a large collection of enveloped viruses (e.g., MoMLV) since VNP bud from the lipid raft regions of the plasma membrane (Figure 2). Decoration of VNP with appropriate immuno-modulatory molecules of choice allows direct and specific interaction of VNP upon co-culture with distinct leukocyte subsets bearing the receptors for such ligands. Incorporation of fluorescent proteins during the assembly and budding process of VNP, e.g., in the form of matrix protein∷green fluorescent protein(MA∷GFP) fusion proteins into the nascent VNP, leading to the formation so called fluorosomes (FS), permits visualization of specific interactions between ligand-decorated VNP and cell populations (leukocytes) expressing the corresponding receptors. Along those lines we could show in proof of principle experiments that interleukin(IL)-2 decorated VNP can distinguish between freshly isolated naïve T cells, lacking high affinity IL-2 receptor (IL-2R) expression on their surface, and *in vitro* and *in vivo* activated CD25^+^ cells by means of flow cytometric analyses [115]. The interaction of IL-2 decorated FS with IL-2R^+^ T cells was highly specific, since it could be blocked dose-dependently by the addition of increasing amounts of soluble IL-2 [115].

In general, leukocyte development and maturation is associated with gross changes in cell surface receptor expression pattern, which is a direct reflection of the changing functional demands a cell type is going through. On a similar note, malignant hematopoetic diseases affecting distinct leukocyte populations sometimes cause considerable changes in the expression pattern of affected cell types. Accordingly, determination of cell surface receptor expression pattern not only allows the identification of leukocytes residing within a certain state of differentiation, activation or maturation but also permits to distinguish normal from malignant cell types. In fact, it is a major objective of modern immunohematology to be able to accurately identify and assign malignantly transformed leukocyte subsets to clinical risk groups forming the basis for state-of-the-art therapeutic intervention procedures [155]. Along those lines the authors could show in proof of principle experiments that IL-2 decorated FS were able to clearly identify malignantly transformed B non-Hodgkin lymphoma (B-NHL) chronic lymphocytic leukemia (CLL) cells obtained from CLL patients. In contrast to the majority of B cells isolated from the peripheral blood of healthy individuals, they can express high levels of the IL-2R *α*-chain, which might contribute to disease progression due to delivery of anti-apoptotic signals in activated CLL cells [156].

The general applicability of the fluorosome principle was demonstrated by generating IL-7 decorated FS, which identified IL-7R^+^ T cells isolated from healthy human individuals. Cytokine binding studies give more accurate information on cytokine receptor composition than evaluation of individual receptor chains of cytokine receptors. In fact, they directly measure cytokine-cytokine receptor binding and not expression of individual components of a receptor, e.g., one cytokine receptor chain.

However, the FS technology is not restricted to the investigation of cytokine/cytokine-receptor interactions but can also be used to identify receptor-ligand interactions of *bona fide* transmembrane immune receptors. Of note, the decoration of FS with CD80 allowed the identification of cytotoxic T lymphocyte antigen (CTLA)-4 positive target cells [115]. In further experiments, it was shown that the FS technology can also be used to investigate multi-subunit molecular interactions. By targeting all three interleukin-2 receptor (IL-2R)-chains (*i.e.*, IL-2R alpha, beta and gamma) onto FS, it was possible to identify IL-2-expressing target cells displaying a membrane-bound form of IL-2 in a concentration dependent manner. One additional attraction of receptor-decorated VNP is the possibility to employ them as ‘cytokine-sinks’ [157] depriving immune cells from excess growth factors thereby modulating their function [115]. More than that, VNP decorated with several different types of ligands can be used to deliver synergistic signals to selected responder cells, e.g., signal 1 and signal 2 to T cells [147]. Importantly, VNP have been considered as potent subunit vaccination platforms as they induce potent B cell-specific immune responses, even when applied via different routes [158,159]. In addition direct activation of macrophages and dendritic cells by filovirus VNP has been described and such VNP represent promising candidate vaccines to protect from Ebola and/or Marburg virus infections [160–162]. Notably, several VNP based vaccines are already clinically used, e.g., from hepatitis B virus [163], human papilloma virus [31,164] and norovirus [165]. Moreover, VNP have been exploited to directly deliver non-transducing mRNA in a dose-controlled and transient fashion to target cells commonly referred to as pseudotransduction [166,167].

**Figure 3.**
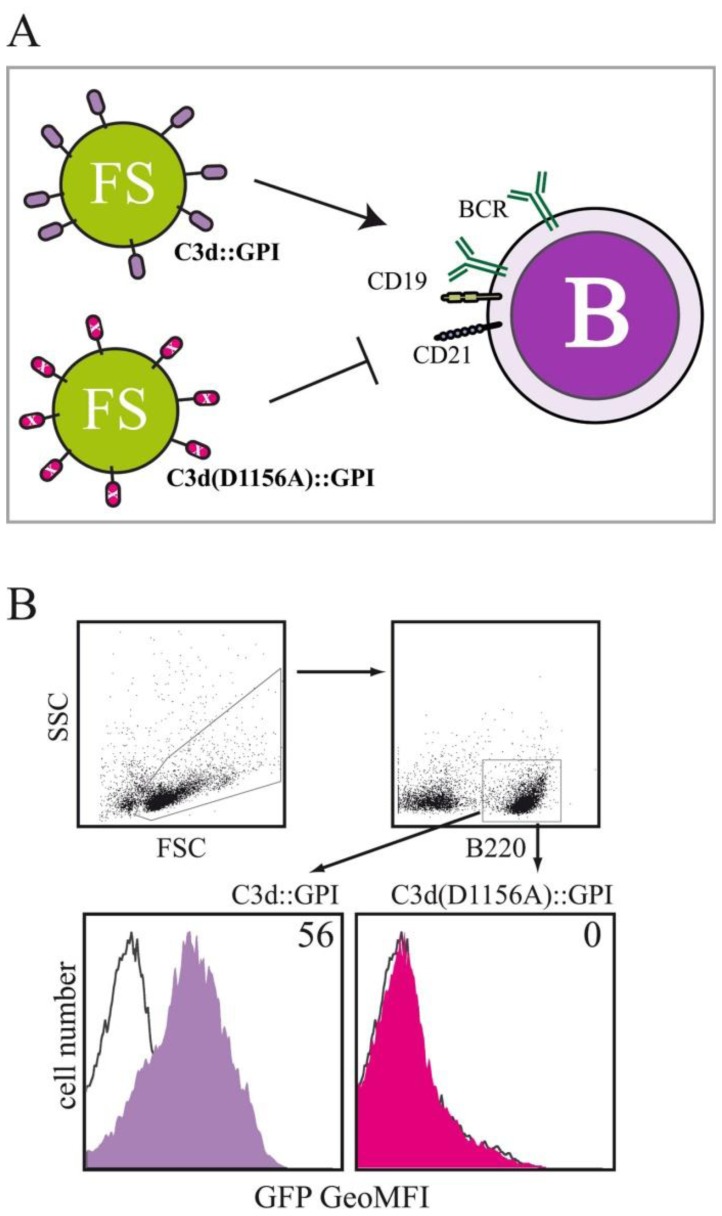
Binding of complement component C3d decorated VNP to B220^+^ murine splenocytes. (**A**) Scheme displaying the two different types of FS. FS decorated with C3d∷GPI can very potently interact with murine B cells. FS decorated with C3d(D1156A)∷GPI fail to interact with murine B cells due to the mutation in the CD21 binding site. (**B**) Splenocytes (1 × 10^6^) obtained from BALB/c mice were incubated with 10 μg MA∷GFP^+^ FS decorated with either C3d∷GPI (filled histogram, lilac), or C3d(D1156A)∷GPI (filled histogram, magenta), or mock decorated FS (solid line black) in the presence of APC conjugated B220^+^ mAb at 4 °C for 60 minutes. B220^+^ lymphocytes (FSC/SSC) were identified (gates indicated) and their GFP fluorenscence (C3d-dependent) displayed as overlay histograms C3d-particles *vs.* mock-particles. Numbers indicate percent positive cells. One representative out of several independent experiments performed is shown.

In order to investigate, whether the fluorosome technology can also be used to study interactions between receptor-ligand pairs belonging to the innate immune system we membrane-anchored the C-terminal fragment of complement component 3, *i.e.*, C3d, a cleavage product of the complement protein C3 with the help of a GPI-anchor attachment sequence. Receptor-ligand-specific interactions between complement receptor 2 (CR2; CD21) positive target cells and C3d-decorated FS were investigated by decorating FS with either C3d∷GPI, the murine C3d fragment fused to the GPI-anchor acceptor sequence of human CD16b, a variant of C3d in which the binding-site for CD21 has been mutated (C3d(D1156A)∷GPI) and full length hCD16b applied for control purposes. Binding of the different types of FS to splenocytes obtained from BALB/c mice was tested by multicolor flow cytometry (Figure 3A). B220^+^ splenocytes (belonging to the B lymphocyte lineage) acquired clear-cut GFP fluorescence when incubated with FS_C3d∷GPI at 4 °C for 60 min (Figure 3B). In marked contrast, FS_C3d(D1156A)∷GPI decorated fluorosomes did not specifically bind to B220^+^ splenocytes when compared to background staining. Background staining was determined by incubating splenocytes with FS decorated with the negative control molecule hCD16b (Figure 3A). Fluorescence intensity values obtained for FS_C3d∷GPI and FS_C3d(D1156A)∷GPI were corrected for background staining and are given as geometric mean fluorescence intensity (GeoMFI) values. Incubation of splenocytes with titrated amounts of differentially decorated VNP followed by flow cytometric analysis allowed determination of half-maximum binding rates (ED_50_). Half-maximum binding rates with FS decorated with C3d∷GPI were obtained by incubating 1 × 10^6^ splenocytes with 8.1 ± 1.8 μg VNP (mean ± SD). In marked contrast FS_C3d(D1156A)∷GPI did not bind to B220^+^ splenocytes (*p* < 0.001, Student's t-test, *n* = 3) at similar concentration (data not shown).

Moreover, the specificity of the C3d-CD21 interaction was assessed by addition of titrated amounts of the CD21 blocking mAb 7G6 [168] which were able inhibit C3d mediated particle binding in a dose dependent manner (not shown). Evaluations, testing the functional consequences of binding of C3d decorated VNP to target cells are ongoing. Previous studies have shown that blocking CD21 monoclonal antibodies or anti sera prevent EBV infection of human B cells [169–171]. Our preliminary results suggest that C3d-decorated VNP function as specific competitors of EBV infection of human B cells (manuscript in preparation).

In summary, fluorescent virus particles have been used in the past to better characterize many aspects of virus biology including mechanisms operative during infection, uncoating, intracellular transport and assembly of virus particles. Moreover, fluorescent virus-like nanoparticles have been established during the last years and represent a powerful tool to visualize receptor-ligand interactions. The lipid-membrane context of FS induced by core-proteins derived from enveloped viruses allows the expression of multi-subunit molecules (receptors) and the identification of target cells expressing the respective interaction partners (ligands). In addition, VNP allow the expression of several copies of one and the same molecule on their surface increasing their avidity for the respective ligand and thus potentially facilitating the visualization of low-affinity interactions. Moreover, the lipid-membrane composition of VNP should allow for the expression and functional evaluation of type III integral membrane proteins, which pass the plasma membrane several times [172] and are associated with lipid rafts [173,174]. The use of novel fluorescent proteins with alternative emission spectra, like the recently described “fruit” colors [105] bears the potential to use several differentially decorated and labeled FS within the same experiment. Apart from studying virus biology, virus particles can also be used as a platform to anchor proteins of interest and to study receptor-ligand interactions. Thus VNP not only represent a useful and versatile tool to study and identify a variety of different receptor-ligand interactions but also possess the capability to very potently modulate functions and fates of target cells of interest.

## Figures and Tables

**Table 1.** Fluorescent labels applied for chemical labeling of viral particles.

**Labeling Method**	**Fluorescent Label**	**Virus/VNP**	**Reference**
**Non-Covalent Linkage**			

lipophilic dyes	**PKH67 (green)** **PKH26 (red)** **DiD/Dil octadecyl rhodamine** **B chloride (R18)**	NDV	[49]
		Influenza A, HIV, Dengue virus, VSV	[50–53]
		Sendai Virus, VSV, HIV, RSV, NDV	[54–59]

Intercalating dye	**[Ru(phen)_2_(dppz)]^2+^**	Baculovirus	[60]

Biarsenical dyes	**FlAsH (Fluorescein)** **ReAsH (Resurfin)**	VSV, FHV, HIV, Ebola Virus	[61–68]

**Covalent Linkage**			

Amine reactive groups	**Fluorescein/FITC**	EBV, Reovirus, HIV, VSV	[69–73]
	**FITC disphophate**	Influenza A	[74]
	**Alexa Fluor488**	AAV, Influenza A, Dengue Virus	[75–77]
	**Cy3**	Ad, AAV	[47,78]
	**Rhodamine derivates**	Ad, Reovirus	[71,79]
	**Alexa Fluor594**	DengueVirus	[77]
	**CypHer5**	Influenza A	[50]
	**Cy5**	AAV	[46]
	**IRDye®800CW**	SHIV VNP	[48]

	**Quantum dots**	HTLV, HAV, MNV, Influenza A, MoMLV, IHNV	[80–84]

AAV (adeno-associated virus), Ad (adenovirus), EBV (Eppstein Barr virus), FHV (flock house virus), FITC (fluorescein isothiocyanate), HAV (hepatitis A virus), HIV (human immunodeficiency virus), HTLV (human T cell leukemia virus), IHNV (infectious hematopoietic necrosis virus), MNV (murine norovirus), MoMLV (moloney murine leukemia virus), NDV (Newcastle disease virus), NIR (near infrared), RSV (respiratory syncytial virus), SHIV (simian immunodeficiency virus), VSV (vesicular stomatitis virus).

**Table 2.** Generation of fluorescent viral particles by genetic fusion of fluorophores to proteins of viral and non-viral origin.

**Virus (VNP)**	Fusion Partner of Viral or Non-Viral (*) Origin	**Fluorophore**	**Application**	**Reference**
**RNA-viruses (enveloped)**				
*Retroviridae*				
**MoMLV**	MA	GFP	investigation of receptor-ligand interactions of non-viral origin	[115]
	SU	GFP	influence of insertions into viral envelope proteins on infectivity	[116]
**HIV**	Gag	eGFP	subcellular Gag localization upon virus assembly	[117]
	Vpr	GFP	intracellular cytoskeleton-dependent trafficking upon infection	[52]
	MA	eGFP	host cell infection	[118]
	Vpr MA	mRFP1 eGFP	double-labeled viral particles for subcellular localization of viral proteins upon infection	[110]
	S15 p60c-Src *	mCherry	virus-host cell fusion	[113]
	GPI-anchor of CD55 *	GFP (monomeric)	fluorescence molecular painting (FMP), virus attachment	[114]
*Orthomyxoviridae*				
**Influenza A**	M2	eGFP	VNP uptake by host cells	[119]
	GPI-anchor of CD55 *	GFP (monomeric)	fluorescence molecular painting (FMP), virus attachment	[114]
*Rhadbdoviridae*				
**RV**	Phosphoprotein P	eGFP	virus binding and internalization, interaction with other viral proteins	[120]
	Glycoprotein G Phosphoprotein P	mRFP (tdtomato) eGFP	fate of envelope and RNP upon viral infection, determination of composition of viral particles transported within cells	[121]
**VSV**	Glycoprotein G	eGFP	influence of GFP on virus assembly, host cell infection	[122]
	Phosphoprotein P	eGFP	dynamic imaging of M protein distribution by dual biarsenical labeling, mechanisms of virus uncoating	[61]
*Paramyxoviridae*				
**Measles virus (MV)**	L-protein	eGFP	localization of L protein within infected cells	[123]
**RNA-viruses (non-enveloped)**				
*Reoviridae*				
**Rotavirus derived VNP**	VP2	GFP	VNP interaction with and entry into living cells	[124]
**DNA-viruses (enveloped)**				
*Poxviridae*				
**Vaccinia virus**	B5R	GFP	intracellular movement, test system to test drugs inhibiting intracellular virus trafficking (anti-viral drug systems)	[125]
	p37	GFP	cytoskeleton dependent virus motility upon assembly	[126]
*Herpesviridae*				
**HSV**	VP26	GFP	infection, localization of capsid protein VP26 upon virus assembly	[127]
	VP22	GFP	subcellular distribution upon virus replication	[128]
	ICP27	eGFP	intracellular localization and trafficking of potential loss-of-function mutants	[129]
	VP16	eGFP	retrograde movement of virus along axons	[130]
			subcellular organization and compartmentalization	[131]
	VP13/14	YFP	subcellular localization upon virus tegument assembly	[132]
	VP11/12	GFP	distribution upon infection, rapid directional, translocations during virus replication	[133]
	VP22 VP13/14	CFP YFP	dynamics of subcellular distribution during virus replication	[109]
	gB	eGFP	subcellular localization upon virus assembly	[134]
	DNA-binding protein (TetR)	eYFP	viral genome localization upon infection	[135]
	ICP4, ICP0	eCFP, eYFP	dynamics of subcellular distribution upon virus assembly	[136]
	VP26	mRFP1	transport of viral capsids in sensory axons	[137]
	VP26 VP22 gB	VenusA206 mRFP ECFPA206K	virus entry and uncoating, Simultaneous observation of capsid, tegument and envelope proteins during virus assembly processes	[111]
**HCMV**	pp150	eGFP	intracellular viral particle movement during virus life (live images)	[138]
**Feline Herpesvirus**	GPI-anchor of CD55 *	GFP (monomeric)	fluorescence molecular painting (FMP), virus attachment	[114]
**Pseudorabies Virus**	gG	GFP	spread of virus between living cells	[139]
	VP26	GFP	viral anterograde transport kinetics along axons of sensory neurons	[140]
	VP26 VP22	mRFP GFP	virus-host interaction, directional transport of viral proteins upon assembly in axons	[108]
*Asfariviridae*				
**African swine fever virus**	p54	eGFP	virus-host infection, intracellular localization upon infection	[141]
*Baculoviridae*				
**Baculovirus**	GP64	GFP	multicolor labeling in combination with a labeled genome, cellular uptake and subcellular distribution	[60]
**DNA-viruses (non-enveloped)**				
*Parvoviridae*				
**AAV**	VP2	eGFP	virus infection, intracellular trafficking upon infection	[142]
	VP1, VP2	GFP	influence of insertion of peptides of different size on particle formation and infectivity	[143]
*Adenoviridae*				
**Ad**	MAP4	eGFP	cytoskeleton dependent intracellular trafficking	[144]
	DNA-binding protein (TetR)	GFP	AdLite particles, intracellular trafficking of viral genomes	[145]

AAV (adeno-associated virus), Ad (adenovirus), A MLV (amphotropic murine leukemia virus), CA (capsid protein), HCMV (human cytomegalovirus), HIV (human immunodeficiency virus), HSV (herpes simplex virus), ICP (infected cell protein), IN (integrase), LV (lentivirus), MA (matrix protein), MAP4 (microtubule-associated protein 4), MoMLV (moloney murine leukemia virus), NC (nuclear capsid protein), PR (protease), RN (RNase-H), RT (reverse transcriptase), RV (rabies virus), TC (tetracysteine), tm (transmembrane).

## List of abbreviations

C3: complement component 3
CD: cluster of differentiation
CLL: chronic lymphocytic leukemia
CR2: complement receptor 2
DNA: deoxyribonucleic acid
ED_50_: effective dose 50 percent
FS: fluorosome
Gag: group specific antigen of retrovirus, *i.e.*, core proteins
GFP: green fluorescent protein
GM-CSF: granulocyte macrophage colony-stimulating factor
GPI: glycosyl phosphatidyl inositol
HIV: human immunodeficiency virus
IL: interleukin
IL-2R: interleukin-2 receptor
MA: matrix protein of retrovirus
MoMLV: moloney murine leukemia virus
PKH: Paul Karl Horan, inventor of PKH dyes
RNA: ribonucleic acid
RFP: red fluorescent protein
SD: standard deviation
VNP: virus-like nanoparticle
VSV: vesicular stomatitis virus
YFP: yellow fluorescent protein
